# 2478. Trends in antibiotic resistance for nine pathogens in the US Veterans Affairs Healthcare System from 2007 to 2022

**DOI:** 10.1093/ofid/ofad500.2096

**Published:** 2023-11-27

**Authors:** Thi Mui Pham, Yue Zhang, McKenna R Nevers, Haojia Li, Karim Khader, Marc Lipsitch, Yonatan H Grad, Matthew H Samore

**Affiliations:** Harvard T.H. Chan School of Public Health, Boston, MA; University of Utah, Salt Lake City, Utah; University of Utah, Salt Lake City, Utah; University of Utah, Salt Lake City, Utah; University of Utah, Salt Lake City, Utah; Harvard T.H. Chan School of Public Health, Boston, MA; Harvard Chan School of Public Health, Boston, Massachusetts; University of Utah, Salt Lake City, Utah

## Abstract

**Background:**

Antibiotic-resistant infections are a major public health concern. To better understand the burden of these infections, we analyzed incidence and antibiotic resistance trends for nine important organisms in the US Veterans Affairs (VA) Healthcare System from February 2007 to March 2022.

Example plot of reporting resistance burden.

**Figure:**
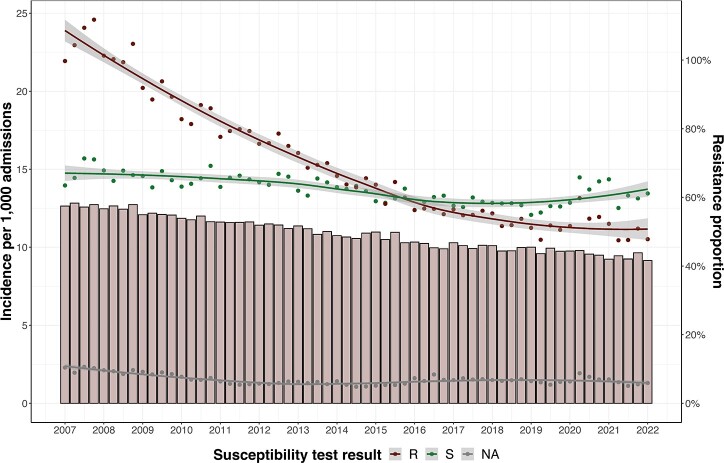

The bar blot represents the proportion of isolates resistant to the respective drug that includes the number of missing test results in the denominator. Green points represent the incidence of isolates susceptible to the drug (S). Red points represent the incidence isolates resistant to the drug (R). Grey points represent the incidence of isolates with missing susceptibility test results (NA). Solid lines and grey bands represent smoothing curves using the loess method and the 95% confidence intervals, respectively.

**Methods:**

We collected microbiology data from all patients admitted to acute-care wards in 158 VA Medical Centers in the US. For nine bacterial pathogens, we evaluated two types of metrics: 1) incidence of resistant or susceptible clinical isolates per 1,000 admissions, and 2) proportion of 30-day incident isolates resistant to a given drug class. Inpatient antibiotic use was calculated as the days of therapy (DOT) divided by 1,000 patient days.

**Results:**

Trends in resistance proportions for beta-lactam antibiotics varied by species and beta-lactam class. The proportion resistant to 3rd and 4th generation cephalosporins increased in *Escherichia coli* isolates (from 7.5% to 18% and 4% to 10%, respectively) but remained stable for *Klebsiella pneumoniae* (∼ 15% and ∼ 8%, respectively). Carbapenem resistance remained low for both pathogens. In contrast, resistance to anti-pseudomonal beta-lactams in *Pseudomonas aeruginosa* and methicillin resistance in *Staphylococcus aureus* declined from 29.5% to 21.1% and from 58% to 41%, respectively. Most species demonstrated trends of decreasing resistance proportion for fluoroquinolones. The decline was associated with an increase in incidence of susceptible isolates for *E. cloacae, E. faecalis, E. coli, and P. aeruginosa*. Trends in inpatient antibiotic use included substantial decreases in fluoroquinolone use (from 125 to 20 DOT per 1,000 patient days), smaller changes in the use of beta-lactam and glycopeptide drugs, and an increase in the use of 3rd and 4th generation cephalosporins (from 48 to 79 and from 19 to 43 DOT per 1,000 patient days).

Fluoroquinolone resistance burden for all nine species from 2007 to 2022.

**Figure:**
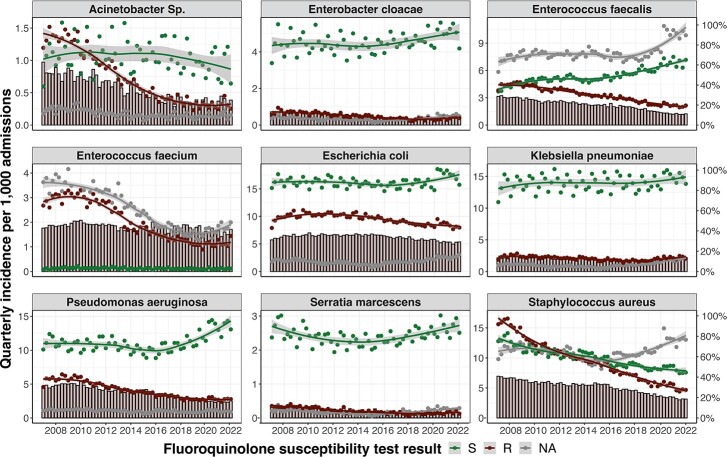

Dark bar plots represent the proportion of isolates resistant to fluoroquinolones where missing values were included in the denominator (right axis). Points represent the incidence rate of the respective fluoroquinolone susceptibility test results. Green points represent the quarterly incidence of isolates susceptible to fluoroquinolones. Red points represent the quarterly incidence isolates resistant to fluoroquinolones. Grey points represent the quarterly incidence of isolates with missing susceptibility test results. Solid lines and grey bands represent smoothing curves using the loess method and the 95% confidence intervals, respectively.

Inpatient antibiotic utilization from patients admitted to Veterans Affairs Medical Centers from 2007 till 2022.

**Figure:**
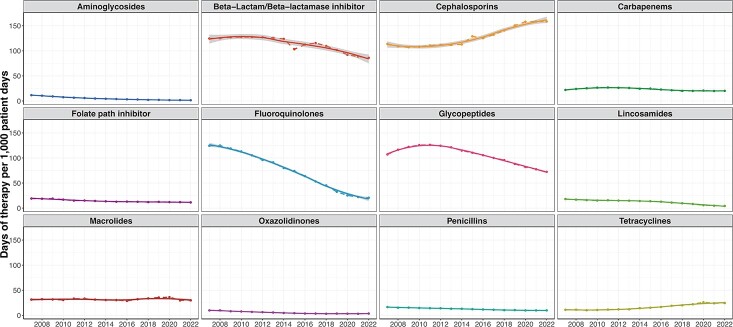

Inpatient antibiotic utilization from patients admitted to Veterans Affairs Medical Centers from 2007 till 2022. Points are days of therapy per 1,000 patient days in each year. Dashed lines are linear interpolations between the points. Solid lines are smoothed curves using the loess method and grey bands represent the respective 95% confidence interval.

**Conclusion:**

The proportion of fluoroquinolone resistant isolates declined for multiple species and is associated with a decline in inpatient fluoroquinolone use, whereas the proportion of beta-lactam resistant isolates varied by class of beta-lactam and by pathogen. Our study highlights the need for continued surveillance and more research to understand these trends.

**Disclosures:**

**Marc Lipsitch, PhD**, Janssen: Advisor/Consultant|Pfizer: Grant/Research Support **Yonatan H. Grad, MD, PhD**, Day Zero Diagnostics: Board Member|GSK: Advisor/Consultant

